# Crystal growth control of rod-shaped ε-Fe_2_O_3_ nanocrystals†

**DOI:** 10.1039/d0ra07256g

**Published:** 2020-10-29

**Authors:** Hiroko Tokoro, Junpei Fukui, Koki Watanabe, Marie Yoshikiyo, Asuka Namai, Shin-ichi Ohkoshi

**Affiliations:** Department of Materials Science, Faculty of Pure and Applied Sciences, University of Tsukuba 1-1-1 Tennodai, Tsukuba Ibaraki 305-8573 Japan tokoro@ims.tsukuba.ac.jp; Department of Chemistry, School of Science, The University of Tokyo 7-3-1 Hongo, Bunkyo-ku Tokyo 113-0033 Japan ohkoshi@chem.s.u-tokyo.ac.jp

## Abstract

Herein we report crystal growth control of rod-shaped ε-Fe_2_O_3_ nanocrystals by developing a synthesis based on the sol–gel technique using β-FeO(OH) as a seed in the presence of a barium cation. ε-Fe_2_O_3_ nanocrystals are obtained over a wide calcination temperature range between 800 °C and 1000 °C. A low calcination temperature (800 °C) provides an almost cubic rectangular-shaped ε-Fe_2_O_3_ nanocrystal with an aspect ratio of 1.4, whereas a high calcination temperature (1000 °C) provides an elongated rod-shaped ε-Fe_2_O_3_ nanocrystal with an aspect ratio of 3.3. Such systematic anisotropic growth of ε-Fe_2_O_3_ is achieved due to the wide calcination temperature in the presence of barium cations. The surface energy and the anisotropic adsorption of barium on the surface of ε-Fe_2_O_3_ can explain the anisotropic crystal growth of rod-shaped ε-Fe_2_O_3_ along the crystallographic *a*-axis. The present work may provide important knowledge about how to control the anisotropic crystal shape of nanomaterials.

## Abstract 2

Crystal growth control of rod-shaped ε-Fe_2_O_3_ nanocrystals is achieved by a synthesis based on the sol–gel technique.

## Graphical abstract

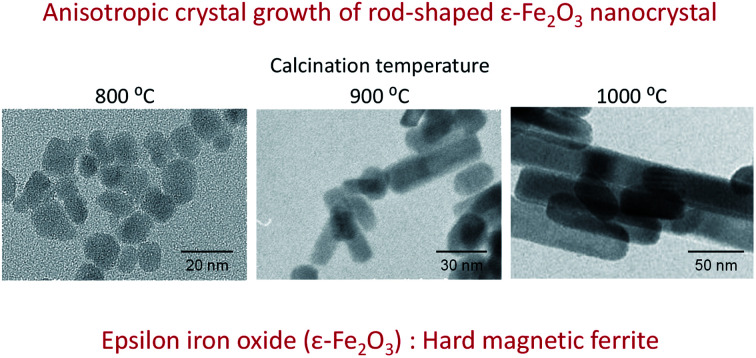

## Introduction

Ferrite magnets are ubiquitous functional materials for industry due to their ferromagnetic properties.^1–6^ Among ferrite magnets, gamma-diiron trioxide (γ-Fe_2_O_3_) has been extensively used due to its soft magnetic functionalities and chemical stability.^7–10^ On the other hand, epsilon-diiron trioxide (ε-Fe_2_O_3_) has drawn increasing attention in recent years.^11–16^ In 2004, a single phase of ε-Fe_2_O_3_, which was artificially synthesized *via* a nanoscale synthesis, displayed a huge magnetic coercive field over 20 kOe at room temperature.^11^ Since then, various fundamental studies have been reported.^17–23^ Furthermore, practical applications such as high-density magnetic recordings and high-frequency electromagnetic (EM) wave absorption have been considered.^24–27^ From a synthetic viewpoint, different methods for ε-Fe_2_O_3_ have been reported, including the reverse-micelle and sol–gel combination, mesoporous SiO_2_ template, pulsed laser deposition (PLD), and chemical vapor deposition (CVD).^17,28–34^

The ε-Fe_2_O_3_ phase is only formed in the nanosize region. Typically, ε-Fe_2_O_3_ nanoparticles have a spherical shape, but rod-shaped crystals can appear under specific conditions.^12^ Rod-shaped ε-Fe_2_O_3_ has received attention from the viewpoint of applications as oriented fibers and probes for magnetic force microscopy.^12,35^ For example, small rod-shaped ε-Fe_2_O_3_ crystals less than a hundred nanometers are attractive for an oriented optical material because light scattering is eliminated. By contrast, elongated rod-shaped ε-Fe_2_O_3_ crystals are desirable as a probe in magnetic force microscopy. Hence, controlling the particle size of rod-shaped ε-Fe_2_O_3_ nanocrystals is an important issue. To date, rod-shaped ε-Fe_2_O_3_ nanocrystals have only been obtained using a combination of reverse-micelle and sol–gel techniques.^12^ However, this method produces ε-Fe_2_O_3_ in a very limited calcination temperature region (*i.e.*, 960–1040 °C), highlighting the difficulty in controlling the particle size. A method to control the particle size of rod-shaped ε-Fe_2_O_3_ has yet to be developed. In this work, we report a synthetic method to prepare rod-shaped ε-Fe_2_O_3_ nanocrystals with a wide range of calcination temperatures to control the particle size and investigate a long-standing problem: the mechanism of anisotropic crystal growth of rod-shaped ε-Fe_2_O_3_.

Here, we report the synthesis of rod-shaped ε-Fe_2_O_3_ nanocrystals based on the sol–gel technique using β-FeO(OH) as a seed with a SiO_2_ matrix. X-ray powder diffraction (XRPD) and Rietveld analyses indicate that ε-Fe_2_O_3_ can be obtained by calcination over a wide temperature range (*i.e.*, 800–1000 °C). Transmission Electron Microscopy (TEM) shows that the size of the rod-shaped crystals is well controlled over a wide range of calcination temperatures. The anisotropic crystal growth of rod-shaped ε-Fe_2_O_3_ can be explained using the surface energy and anisotropic adsorption of barium on the crystal surface.

## Materials and methods

### Materials

**Fig. 1:**
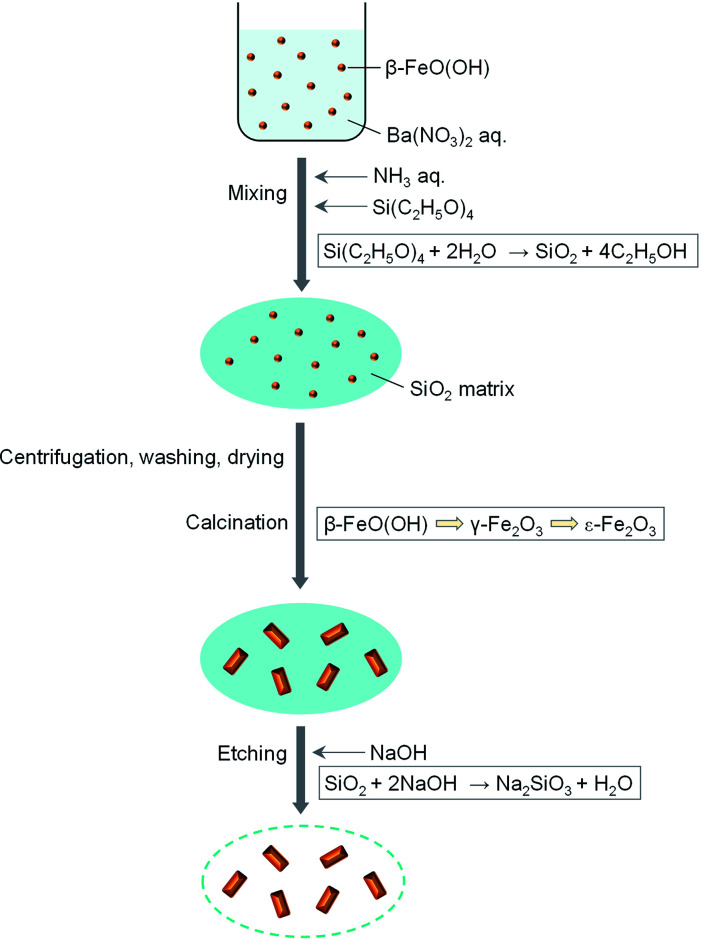
Schematic of the synthesis for rod-shaped ε-Fe_2_O_3_ nanocrystals.

Fig. 1 schematically illustrates the synthesis of rod-shaped ε-Fe_2_O_3_ nanocrystals based on the sol–gel technique using β-FeO(OH) as a seed with a SiO_2_ matrix. Barium nitrate (Ba(NO_3_)_2_) (0.5 g) was added to water-dispersible β-FeO(OH) (4 g) solution (420 mL) (Taki Chemical; Fe–C10). An aqueous solution of 25% ammonia (19.2 mL) was added to a β-FeO(OH) dispersed solution. The mixture was stirred at 50 °C for 30 min. Then tetraethoxysilane (TEOS, Si(C_2_H_5_O)_4_) (24 mL) was added, and the solution was stirred at 50 °C for 20 h. The resultant orange gel was collected by centrifugation, washed with water, and dried at 60 °C for 1 day. The obtained orange powder was calcinated at 800–1000 °C for 4 h in air. The calcinated powder was etched with a NaOH aqueous solution at 70 °C to remove the SiO_2_ matrix that covered the iron oxide nanocrystals. The etched powder was washed with hydrochloric acid. In this work, we prepared 7 samples calcinated at different temperatures: 800 °C (1), 850 °C (2), 875 °C (3), 900 °C (4), 950 °C (5), 975 °C (6), and 1000 °C (7).

### Physical measurements

XRPD patterns were measured by a Rigaku Ultima IV using Cu Kα (*λ* = 1.5418 Å). Rietveld analyses for the XRPD patterns were performed using Rigaku PDXL software. TEM images were acquired using a JEOL JEM 2000EX. TEM energy-dispersive X-ray spectroscopy (TEM-EDX) was performed using a JEOL JEM 2010F. Raman spectra were measured by NRS-5500 Laser Raman Spectrometer (JASCO Corporation, Japan). Magnetic measurements were performed using a Quantum Design MPMS superconducting quantum interference device (SQUID) magnetometer.

## Results and discussion

### Crystal structure, morphology, and magnetic properties of the nanocrystals

**Fig. 2:**
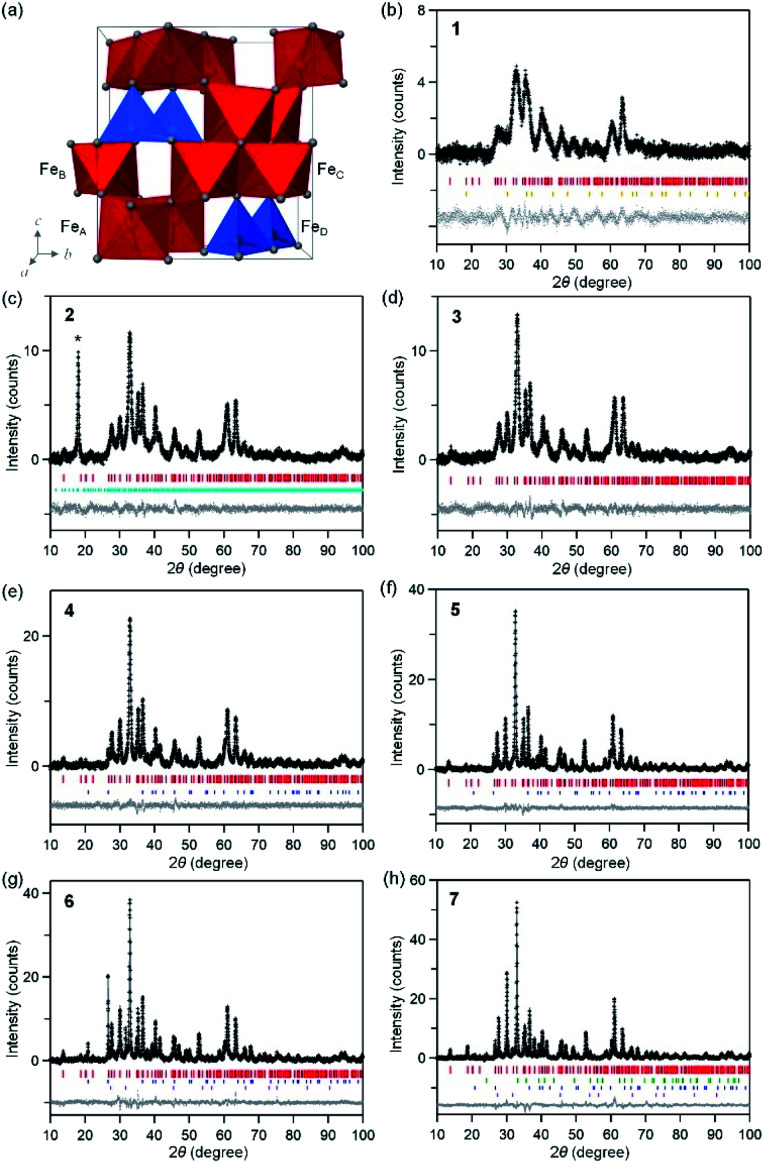
(a) Crystal structure of the orthorhombic unit cell of ε-Fe_2_O_3_. Red and blue polyhedrals in the crystal structure indicate octahedral and tetrahedral Fe sites, respectively. Gray balls represent oxygen atoms. XRPD patterns and Rietveld analyses for 1 (b), 2 (c), 3 (d), 4 (e), 5 (f), 6 (g), and 7 (h). Black dots, gray lines, and gray dots are the observed patterns, calculated patterns, and their differences, respectively. Colored bars indicate the calculated Bragg reflection positions for ε-Fe_2_O_3_ (red), γ-Fe_2_O_3_ (orange), α-Fe_2_O_3_ (green), and a slight amount of impurities of Na_2_Si_4_O_8_ (light blue), SiO_2_ (blue), and NaCl (purple). Asterisk in (c) indicates the peak of Na_2_Si_2_O_8_.

Fig. 2 shows the XRPD patterns with Rietveld analyses of the obtained samples. Sample 1 contains 97.2% of ε-Fe_2_O_3_ with an orthorhombic crystal structure in the *Pna*2_1_ space group and lattice constants of *a* = 5.064(8) Å, *b* = 8.729(14) Å, and *c* = 9.610(9) Å (Fig. 2b and S1, Table S1†). In addition, a slight amount of γ-Fe_2_O_3_ with a cubic crystal structure in the *Fd*3̄*m* space group and a lattice constant of *a* = 8.299(4) Å) is present. Samples 2–6 consist of ε-Fe_2_O_3_ as a single phase of iron oxide (Fig. 2c–g). In sample 7, ε-Fe_2_O_3_ is the dominant phase but a slight amount of α-Fe_2_O_3_ with a hexagonal crystal structure in the *R*3̄*c* space group and lattice constants of *a* = 5.0353(9) Å and *c* = 13.752(3) Å is generated as a nominal phase (Fig. 2h). Fig. S2† shows the phase fraction *versus* temperature in a phase diagram for iron oxide. A single-phase of ε-Fe_2_O_3_ is obtained in a wide calcination temperature range. In the low calcination temperature region, a slight amount of γ-Fe_2_O_3_ appears. On the other hand, α-Fe_2_O_3_ is generated as a nominal phase in the high calcination temperature region. This phase diagram is consistent with the previous study, which reported that ε-Fe_2_O_3_ is generated as a stable intermediate phase between γ-Fe_2_O_3_ and α-Fe_2_O_3_.^29^

**Fig. 3:**
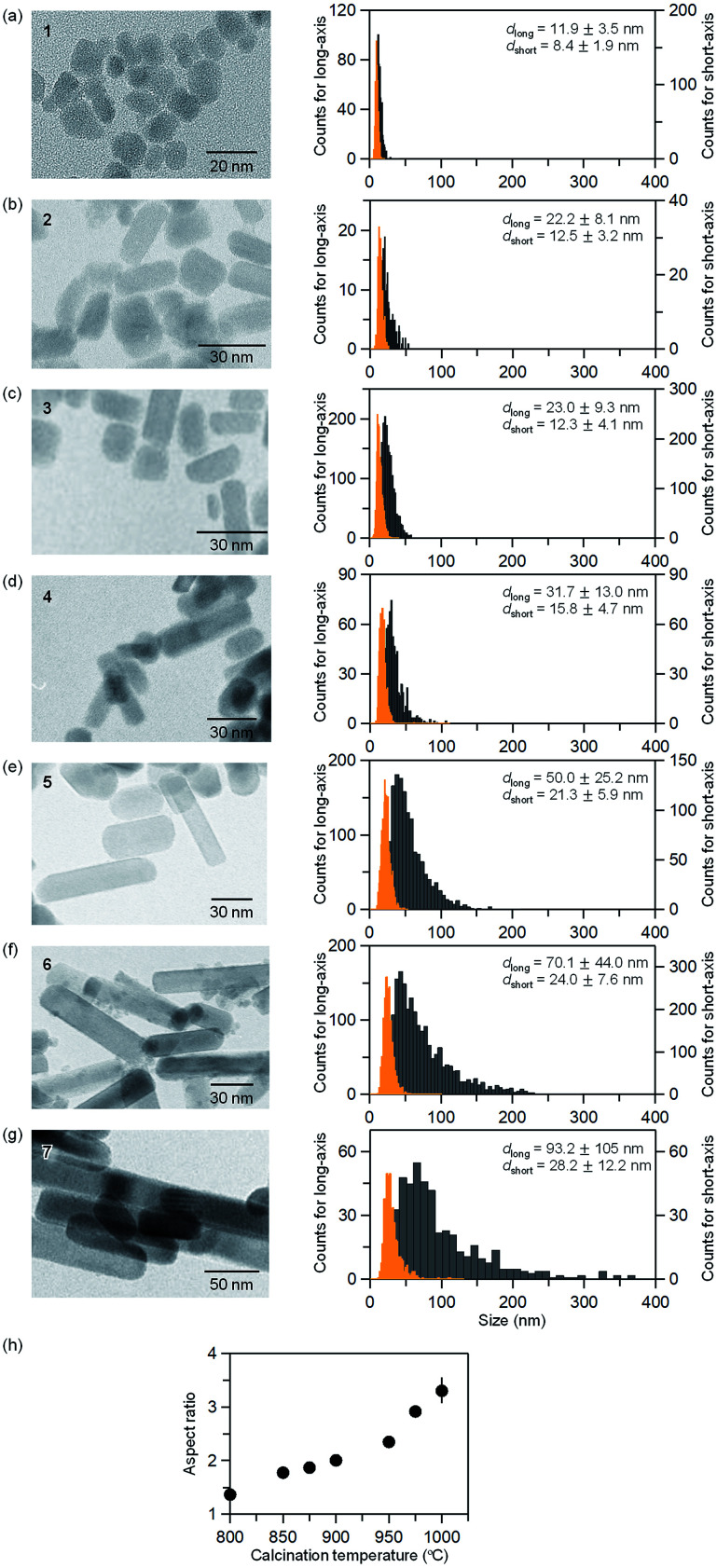
TEM images for samples (a) 1, (b) 2, (c) 3, (d) 4, (e) 5, (f) 6, and (g) 7 (left). Particle size distributions of the long-axis (*d*_long_) (gray) and the short-axis (*d*_short_) (orange) for 1–7 (right). (h) Calcination temperature dependence of the aspect ratio estimated from the average size obtained by the TEM images.

Fig. 3 and Table S2† show the TEM images of the samples and the obtained particle size distributions. In sample 1, the nanoparticles have an almost cubic shape with average sizes of 11.9 ± 3.5 nm for the long axis and 8.4 ± 1.9 nm for the short axis. The particle size distribution follows a log-normal distribution (Fig. S3†). The aspect ratio (the long axis to the short axis) of 1 is 1.4. In sample 2, the average sizes of the long and short axes are 22.2 ± 8.1 nm and 12.5 ± 3.2 nm, respectively. The aspect ratio is 1.8. As the calcination temperature increases, in samples 3–7, the size of the long axis abruptly increases while the size of the short axis gradually increases (*i.e.*, the aspect ratio increases as shown in Fig. 3h). In sample 7, the nanoparticle has an elongated shape, and the average sizes of the long and short axes are 93.2 ± 105 nm and 28.2 ± 12.2 nm, respectively. This gives an aspect ratio of 3.3. As the calcination temperature increases, the long axis grows, leading to an elongated rod-shape. In the case of the reported spherical-shaped particles, changing the calcination temperature controls the size. For example, the diameter is 5.5 ± 1.6 nm at 902 °C, 5.6 ± 1.6 nm at 951 °C, and 7.8 ± 2.7 nm at 1002 °C.^32^ The particle sizes are considerably smaller than the rod-shaped ε-Fe_2_O_3_ synthesized by the present method. This difference indicates that barium promotes crystal growth of ε-Fe_2_O_3_.

Fig. S4† shows the magnetic hysteresis loops at room temperature up to 7 Tesla. The magnetic coercive field (*H*_c_) value increases as the calcination temperature increases (*i.e.*, the particle size increases). This observed trend corresponds to the reported trend where *H*_c_ increases as the particle size increases because the superparamagnetic effect operates in a small particle region.^32,36,37^ The magnetization values for 1–5 become higher as the calcination temperature increases. However, in 6 and 7, the magnetization values are low, even though the calcination temperature is high. One possible reason for the low magnetization is the formation of a slight amount of α-Fe_2_O_3_ (Fig. S5†), which is not observed in the XRD measurement.^38^ Another reason, it is speculated that when the particle shape becomes elongated into a rod, the particles are likely to overlap with each other due to the magnetic force. The rod-shaped ε-Fe_2_O_3_ particle is confirmed to be a single domain magnet.^35^ In such a case, the magnetic moments of ε-Fe_2_O_3_ particles may cancel each other. Consequently, the net total magnetization becomes smaller.

### The mechanism of anisotropic crystal growth

**Fig. 4:**
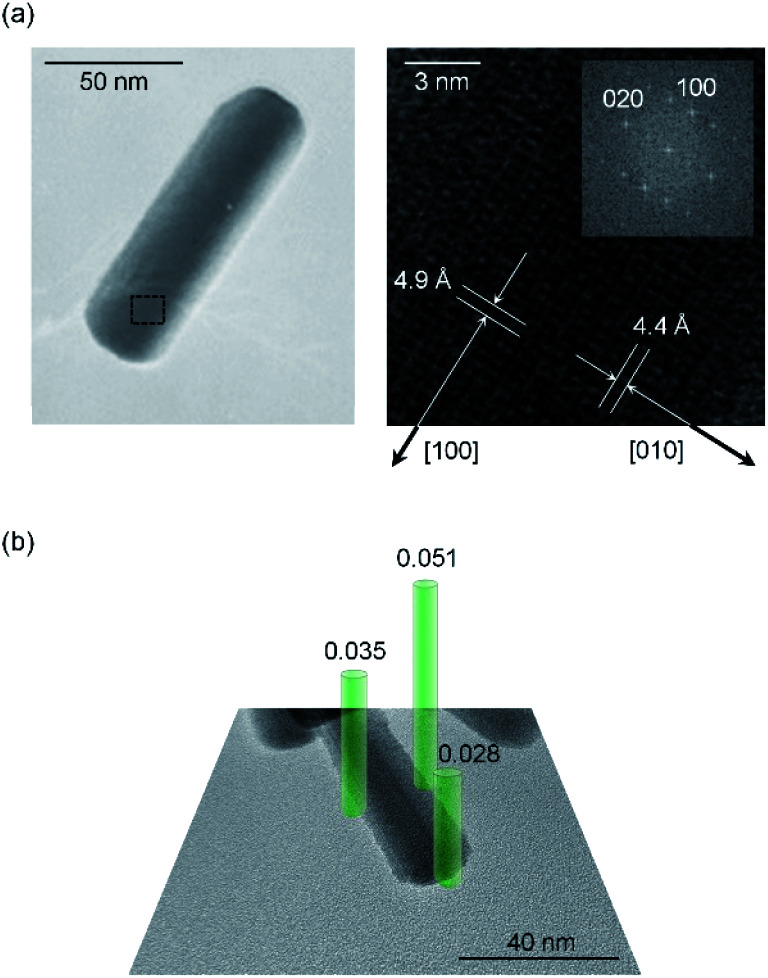
(a) TEM image of 7. Rod-shaped ε-Fe_2_O_3_ shows that the longitudinal direction is along the crystallographic *a*-axis of ε-Fe_2_O_3_ ([100] direction). Right figure is an enlarged view of the dotted-line frame in the left figure. Inset shows the Fourier transformed image. (b) TEM image of 6 showing the Ba concentrations of different areas on the rod-shaped ε-Fe_2_O_3_ (green bars). Ba concentrations are measured by EDX and calculated as the molar ratio with respect to the Fe_2_O_3_ formula. Green bar diameters indicate the spot sizes of the EDX measurement.

Next, the origin of high anisotropic crystal growth of rod-shaped ε-Fe_2_O_3_ is examined. The TEM images show that the ε-Fe_2_O_3_ particle is a single crystal and the longitudinal direction of rod-shaped ε-Fe_2_O_3_ is the crystallographic *a*-axis (*i.e.*, the [100] direction) (Fig. 4a).^39^ Hence, rod-shaped ε-Fe_2_O_3_ nanocrystal is a rectangular-type rod with the long axis in the [100] direction (Fig. S6†).

**Fig. 5:**
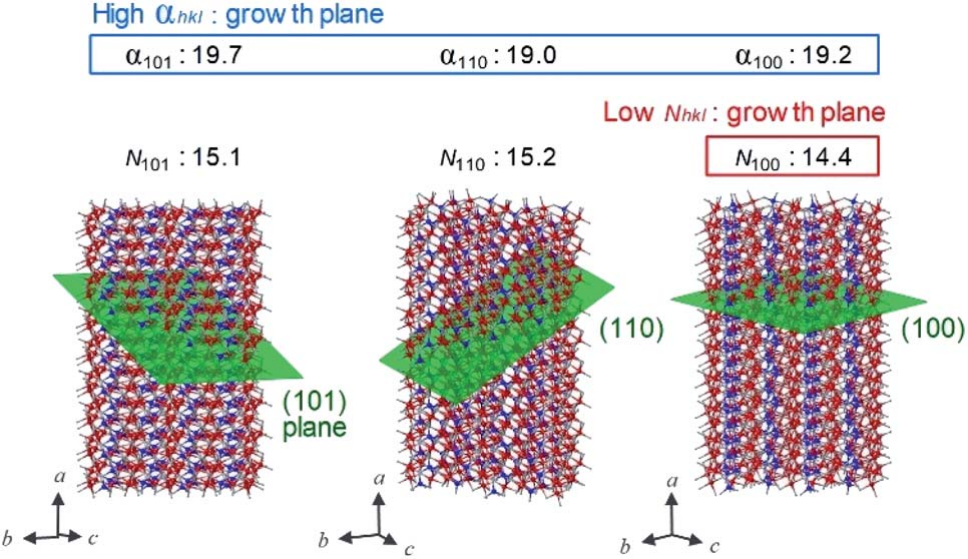
Schematic of the lattice planes (green plane), values of surface energy (*α*_*hkl*_, nm^−2^), and number of FeO_6_-broken bond per area (*N*_*hkl*_, nm^−2^) for the rod-shaped ε-Fe_2_O_3_ nanocrystals (Table S3†). Red, blue, and gray spheres in the crystal structure indicate the Fe at octahedral site, Fe at tetrahedral site, and O, respectively. Large *α* and small *N* values indicate the easy-growth plane.

To understand the anisotropic crystal growth of the nano-rod along the [100] direction, we calculated the surface energies of the (*hkl*) planes, *α*_*hkl*_, based on the binding energy of each surface. *α*_*hkl*_ is expressed by *α*_*hkl*_ = Σ*E*_*hkl*_/*S*_*hkl*_, where Σ*E*_*hkl*_ is the sum of the binding energies of the broken bonds at the (*hkl*) plane and *S*_*hkl*_ is the area of the lattice plane.^40^ Assuming equal binding energies between all Fe and O sites, the magnitude of the surface energies for typical lattice planes are calculated as: 19.2 nm^−2^ (100), 18.7 nm^−2^ (010), 13.4 nm^−2^ (001), 19.0 nm^−2^ (110), 11.9 nm^−2^ (011), 19.7 nm^−2^ (101), and 17.8 nm^−2^ (111) (Table S3†). The (100), (101), and (111) planes possess high surface energies (Fig. 5), whereas the (001) and (011) planes show low surface energies (Fig. S7†). Hence, the (100), (101), and (111) planes are candidates for the growth crystallographic planes.

Next, we focused on the barium adsorption effect. Fig. 4b shows the results of EDX measurement in TEM to investigate the distribution of the barium around the particles. The green cylinders indicate the areas of the EDX measurements. The calculated molar ratios of barium for each area with respect to the Fe_2_O_3_ formula indicate larger values at the longitudinal side of the rods compared to the edge of the short axis. These results suggest that a larger amount of barium is adsorbed on the longitudinal side of the rod than on the edge surface of the growth direction. We considered the mechanism of anisotropic crystal growth of ε-Fe_2_O_3_ from the viewpoint of anisotropic adsorption of barium on the surface of ε-Fe_2_O_3_ particles. In the calcination process, melting SiO_2_ matrix (Si^4+^ and O^2−^) and Ba^+^ ion exist around ε-Fe_2_O_3_ particles. Since Ba^+^ ion takes an octahedral BaO_6_ coordination geometry with oxygen,^41,42^ barium should be adsorbed on the surface of ε-Fe_2_O_3_ in the form of pseudo-octahedral BaO_6_. On the other hand, on the surface of ε-Fe_2_O_3_ particles, there are two kinds of broken bonds (*i.e.*, broken octahedral FeO_6_ and broken tetrahedral FeO_4_). Since BaO_6_ should be adsorbed at the octahedral FeO_6_ site rather than at the tetrahedral FeO_4_ site, a surface containing a large amount of FeO_6_-broken bonds should be covered with adsorbed barium, suppressing crystal growth (*i.e.*, the growth surface should be the one with a small amount of FeO_6_-broken bonds). We estimated the number of FeO_6_-broken bonds per area, *N*_*hkl*_. Fig. 5 and Table S3† show that the *N*_*hkl*_ values are 14.4 nm^−2^, 15.2 nm^−2^, and 15.1 nm^−2^ for (100), (110), and (101) planes, respectively. *N*_100_ shows the lowest value. These values indicate that the [100] direction, along the *a*-axis, can be the crystal growth direction because barium is less adsorbed on the surface of the (100) plane compared to other planes.

## Conclusions

Rod-shaped ε-Fe_2_O_3_ nanocrystals are synthesized based on the sol–gel technique using β-FeO(OH) as a seed in the presence of a barium cation. In this method, changing the calcination temperature can control the size of rod-shaped ε-Fe_2_O_3_ nanocrystals. A low calcination temperature provides almost cubic rectangular-shaped ε-Fe_2_O_3_ nanocrystals, whereas a high calcination temperature provides elongated rod-shaped ε-Fe_2_O_3_ nanocrystals. Although the mechanism of anisotropic crystal growth of rod-shaped ε-Fe_2_O_3_ has been a long-standing issue, in this work, the surface energy and the anisotropic adsorption of barium on the surface of ε-Fe_2_O_3_ can explain the anisotropic crystal growth of the rod-shaped ε-Fe_2_O_3_ along the crystallographic *a*-axis. These findings should realize hard magnetic ferrite ε-Fe_2_O_3_ nanocrystals for practical applications and provide important knowledge about how to control the anisotropic crystal shape of the nanomaterials.

## Conflicts of interest

The authors declare no competing financial interest.

## Supplementary Material

RA-010-D0RA07256G-s001
