# Supplementary material for: Crystal growth control of rod-shaped ε-Fe2O3 nanocrystals
Source: RSC Adv. 2020 Oct 29;10(65):39611–6. doi: 10.1039/d0ra07256g (PMC9057426; doi:10.1039/d0ra07256g)
Supplement: RA-010-D0RA07256G-s001 [file RA-010-D0RA07256G-s001.pdf]

Electronic Supplementary Material (ESI) for RSC Advances.
This journal is © The Royal Society of Chemistry 2020

Electronic Supplementary Information

Crystal growth control of rod-shaped ε-Fe_2O_3 nanocrystals

Hiroko Tokoro,[1,2,*] Junpei Fukui,[1] Koki Watanabe,[1] Marie Yoshikiyo,[2] Asuka Namai,[2] and Shin-ichi Ohkoshi[2,*]

[1] *Department of Materials Science, Faculty of Pure and Applied Sciences, University of Tsukuba, 1-1-1 Tennodai, Tsukuba, Ibaraki 305-8573, Japan*
[2] *Department of Chemistry, School of Science, The University of Tokyo, 7-3-1 Hongo, Bunkyo-ku, Tokyo 113-0033, Japan*

e-mail: tokoro@ims.tsukuba.ac.jp, ohkoshi@chem.s.u-tokyo.ac.jp

Contents **Page**

§1. Crystal structure analyses. ---------- **Fig. S1** S2
Table S1 S2
Fig. S2 S3
§2. Particle sizes. ---------- **Fig. S3, Table S2** S4,S5
§3. Magnetic hysteresis loops at room temperature. ---------- **Fig. S4** S6
§4. Raman spectra. ---------- **Fig. S5** S7
§5. Rectangular-type rod-shaped ε-Fe_2O_3 crystal and its crystallographic direction. **Fig. S6** S8
§6. Surface energy in ε-Fe_2O_3. ---------- **Table S3** S9
§7. Schematic of the lattice planes, values of surface energy, and number of FeO_6-broken bond per area. ---------- **Fig. S7** S10

§1. Crystal structure analyses.

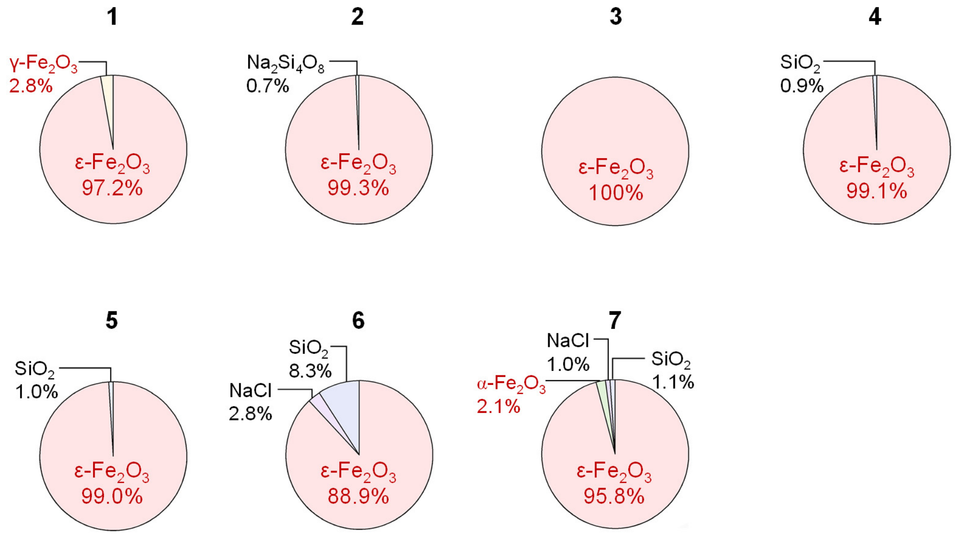

Fig. S1. Phase fractions obtained by Rietveld analyses for samples **1**–**7**.

Table S1. Lattice parameters of ε-Fe_2O_3 in **1**–**7** and refinement parameters obtained by Rietveld analyses of the XRPD patterns.

Sample	Orthorhombic : *Pna* 2_1			Crystal size (nm)	Refinement parameters	
	Lattice parameters					
	a (Å)	*b* (Å)	*c* (Å)		R_{wp} (%)	*S*
**1**	5.064(8)	8.729(14)	9.610(9)	4.4	0.77	1.30
**2**	5.087(2)	8.787(3)	9.489(3)	5.9	0.71	1.17
**3**	5.0906(12)	8.7777(19)	9.4924(15)	5.5	0.75	1.29
**4**	5.0864(4)	8.7856(8)	9.4741(5)	9.8	0.62	1.21
**5**	5.0895(5)	8.7860(10)	9.4705(9)	15.2	0.74	1.29
**6**	5.0896(5)	8.7873(9)	9.4714(9)	16.8	0.88	1.65
**7**	5.0927(3)	8.7859(5)	9.4732(5)	26.6	1.17	1.99

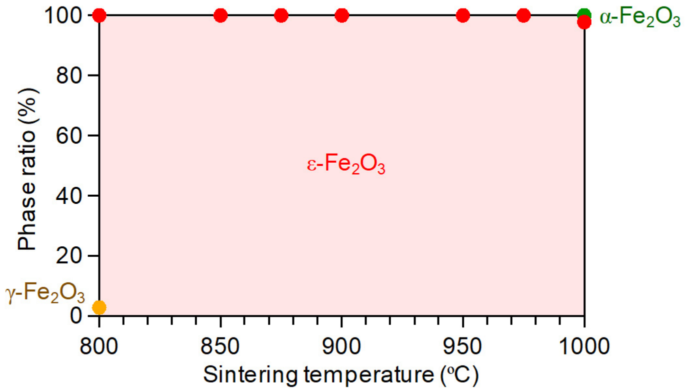

Fig. S2. Phase diagram of the phase ratio versus the sintering temperature for iron oxides.

§2. Particle sizes.

Table S2. Particle size of **1**–**7** obtained by the TEM images.

Sample	Particle size for long axis	Particle size for short axis	Aspect ratio
	d_{long} (nm)	d_{short} (nm)	d_{long} / d_{short}
**1**	11.9 ± 3.5	8.4 ± 1.9	1.4
**2**	22.2 ± 8.1	12.5 ± 3.2	1.8
**3**	23.0 ± 9.3	12.3 ± 4.1	1.9
**4**	31.7 ± 13.0	15.8 ± 4.7	2.0
**5**	50.0 ± 25.2	21.3 ± 5.9	2.3
**6**	70.1 ± 44.0	24.0 ± 7.6	2.9
**7**	93.2 ± 105	28.2 ± 12.2	3.3

The values of the errors are the standard deviation.

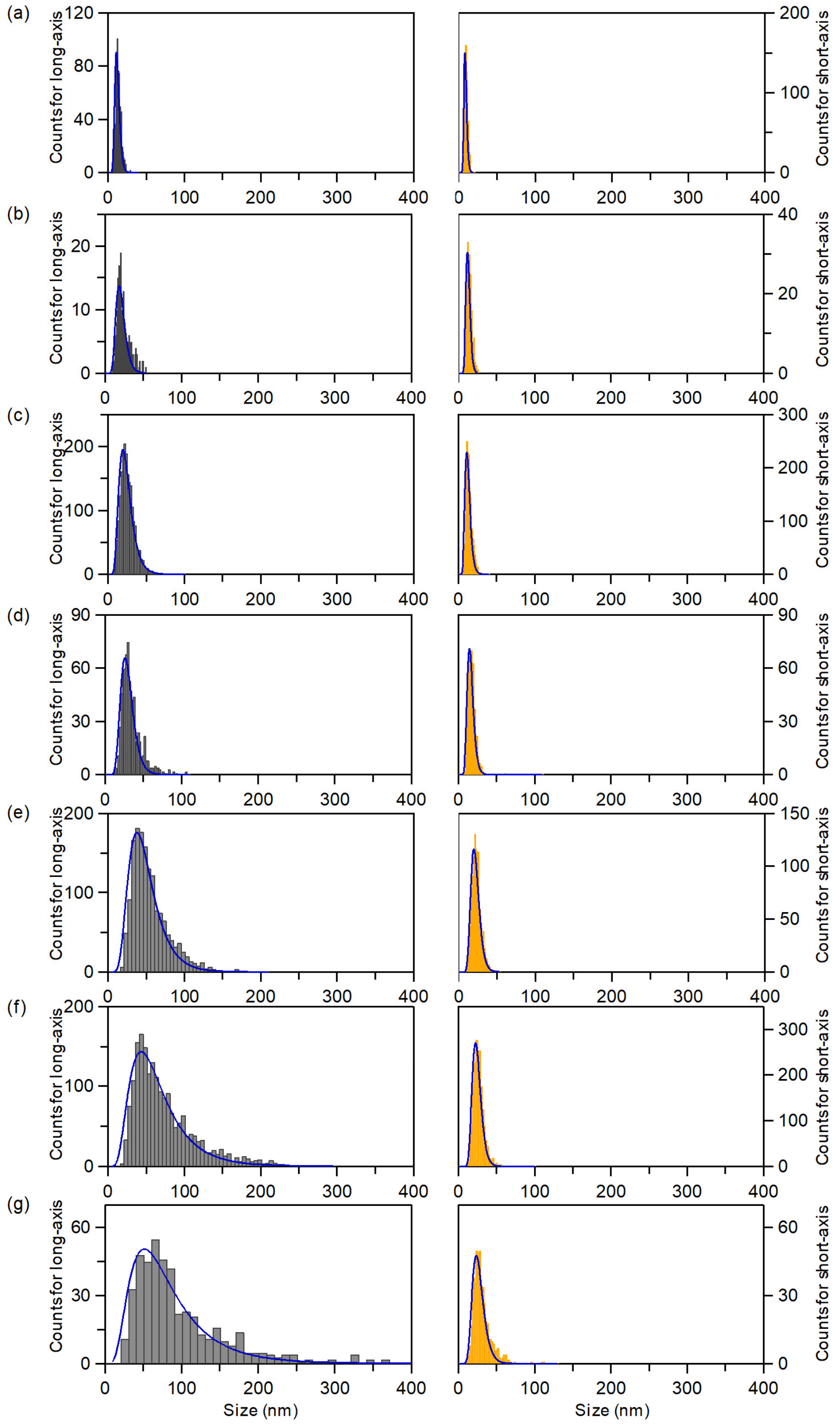

Fig. S3. Particle size distributions of the long-axis (gray) and the short-axis (orange) for **1** (a), **2** (b), **3** (c), **4** (d), **5** (e), **6** (f), and **7** (g). Blue lines are the fitted curve by the log-normal distribution function.

§3. Magnetic hysteresis loops at room temperature.

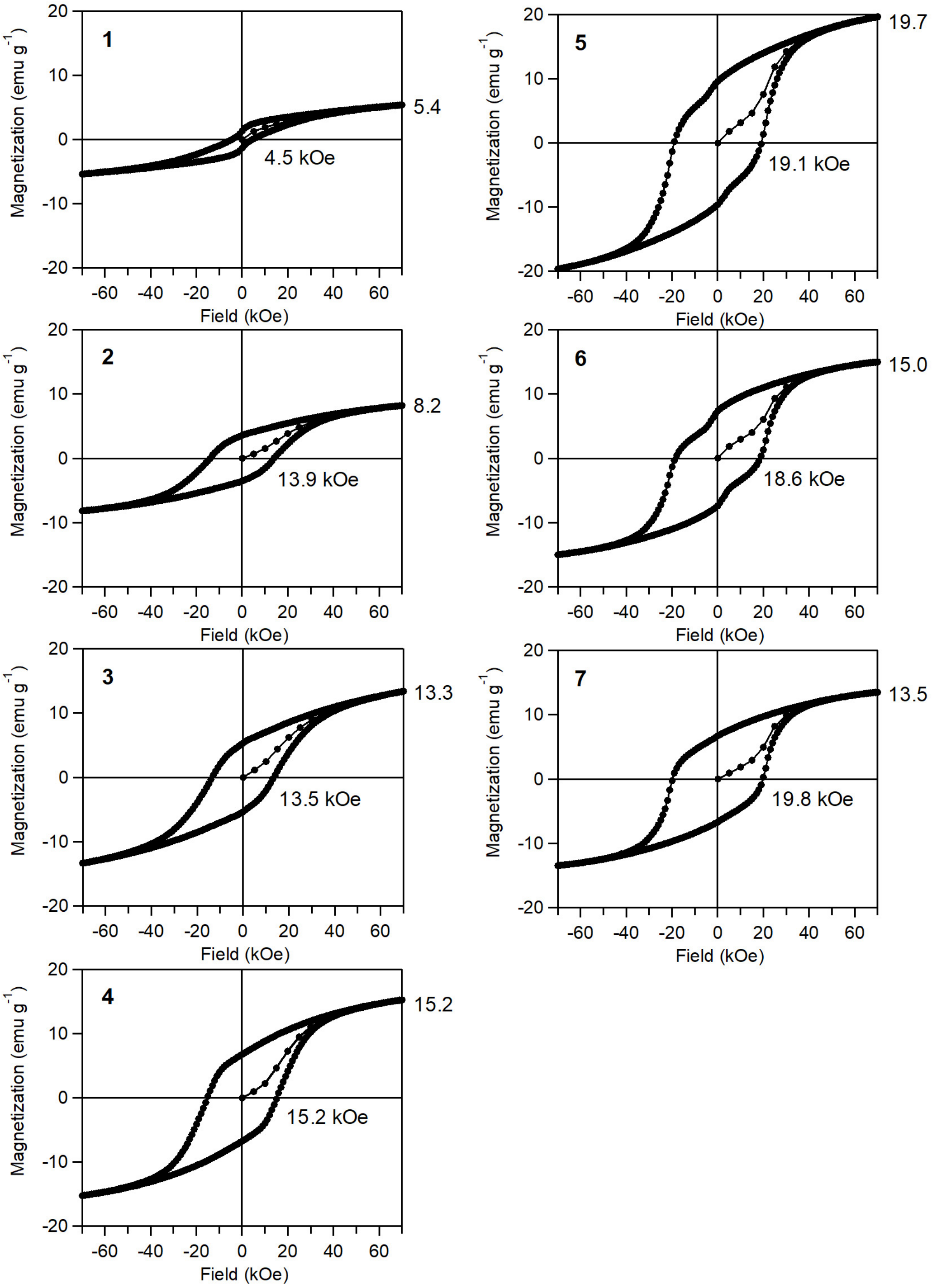

Fig. S4. Magnetic hysteresis loops measured at room temperature up to 7 Tesla for **1**–**7**.

§4. Raman spectra.

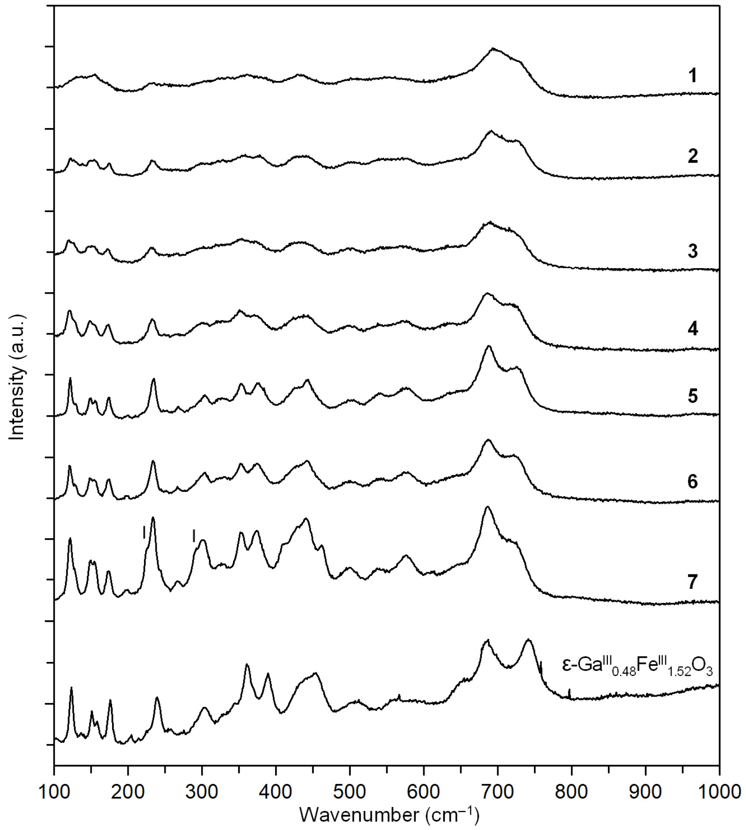

Fig. S5. Raman spectra measured at room temperature for **1**–**7**. Black lines indicate the reported peak positions of α-Fe_2O_3.[38] Bottom spectrum is a reference of ε-$Ga^{III}_{0.48}Fe^{III}_{1.52}O_3$, which was previously reported.[S1] Spectra of **1**–**7** do not show any peaks attributed to the divalent iron. (i.e., Raman spectra of **1**–**7** indicate iron is in the trivalent state).

[S1] S. Ohkoshi, M. Yoshikiyo, Y. Umeta, M. Komine, R. Fujiwara, H. Tokoro, K. Chiba, T. Soejima, A. Namai, Y. Miyamoto, T. Nasu, Phonon mode calculation, Far- and Mid-infrared, and Raman spectra of ε-$Ga_{0.5}Fe_{1.5}O_3$, *J. Phys. Chem. C*, 2017, **121**, 5812–5819.

§5. Rectangular-type rod-shaped ε-Fe_2O_3 nanocrystal and its crystallographic direction.

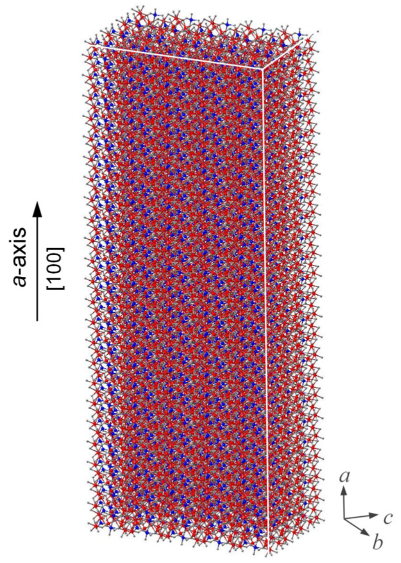

Fig. S6. Schematic of a rectangular-type rod-shaped ε-Fe_2O_3 nanocrystal and its crystallographic direction. Red, blue, and gray spheres indicate Fe at octahedral sites, Fe at tetrahedral sites, and O, respectively.

§6. Surface energy in ε-Fe_2O_3.

Table S3. Surface energy and the ratio of the coordination bonds to the broken bonds in ε-Fe_2O_3.

Lattice plane	Surface energy α_{hkl} (nm^{-2})*	N_{hkl} Number of FeO_6-broken bond per area (nm^{-2})
(1 0 0)	19.2	14.4
(0 1 0)	18.7	16.6
(0 0 1)	13.4	8.9
(1 1 0)	19.0	15.2
(0 1 1)	11.9	7.1
(1 0 1)	19.7	15.1
(1 1 1)	17.8	13.4

*Relative surface energy, number of broken bonds per area assuming that the Fe and O sites have equal binding energies.

§7. Schematic of the lattice planes, values of surface energy, and number of FeO_6-broken bond per area.

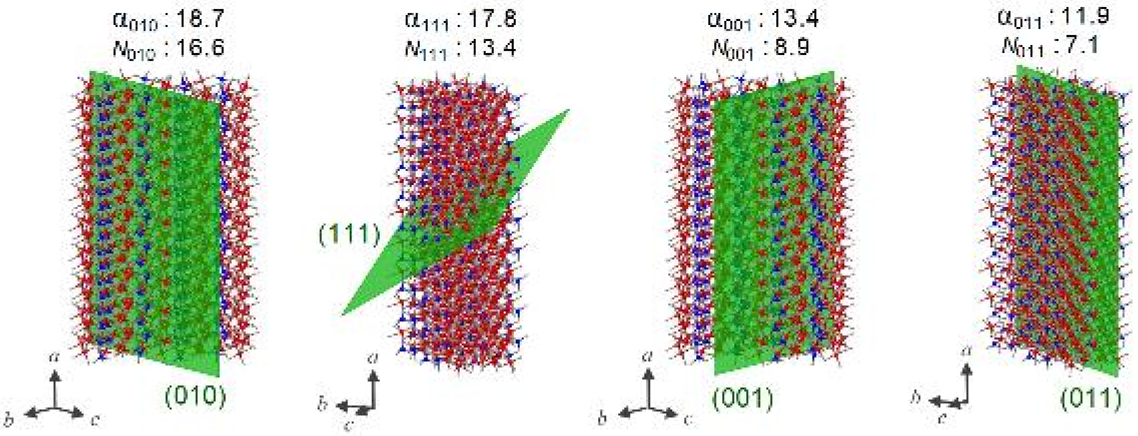

Fig. S7. Schematic of the lattice planes (green plane), values of surface energy (α_{hkl}, nm^{-2}), and number of FeO_6-broken bond per area (N_{hkl}, nm^{-2}) for the rod-shaped ε-Fe_2O_3 nanocrystals. Red, blue, and gray spheres in the crystal structure indicate the Fe at octahedral site, Fe at tetrahedral site, and O, respectively.
